# Determinants of the Cost of Illness in Iranian Prostate Cancer Patients

**DOI:** 10.1155/proc/2149782

**Published:** 2026-04-15

**Authors:** Ramin Ravangard, Khosro Keshavarz, Mozhgan Seif, Shahryar Zeighami, Abdosaleh Jafari, Faride Sadat Jalali

**Affiliations:** ^1^ Health Human Resources Research Center, School of Health Management and Information Sciences, Shiraz University of Medical Sciences, Shiraz, Iran, sums.ac.ir; ^2^ Non-Communicable Diseases Research Center, Department of Epidemiology, School of Health, Shiraz University of Medical Sciences, Shiraz, Iran, sums.ac.ir; ^3^ Department of Urology, School of Medicine, Shiraz University of Medical Sciences, Shiraz, Iran, sums.ac.ir

**Keywords:** cost driver, cost of illness, Iran, prostate neoplasm

## Abstract

**Background:**

Prostate cancer (PCa) ranks as the fourth most prevalent form of cancer overall and imposes a substantial financial burden on individuals and communities. Given information on the costs of this disease and the factors influencing them, the present study analyzed factors affecting the costs of medical and nonmedical services for patients with PCa in southern Iran.

**Methods:**

Factors affecting the cost of PCa were identified through a scoping review of international databases (ISI Web of Science, Scopus, PubMed, ProQuest, Magiran, and SID) and consultation with PCa experts. Subsequently, data on 254 PCa patients were collected between March 2020 and March 2022 using a two‐stage stratified random sampling method. Patients were randomly selected from the provincial Cancer Registry Center lists within strata defined by healthcare catchment areas. Multiple regression analysis using SPSS 13.0 was employed to determine the factors influencing costs.

**Results:**

The findings from the multiple regression analysis indicated that premature mortality, grade, and monthly income (all *p* value < 0.001) were important factors affecting PCa patients’ costs.

**Conclusions:**

In line with the results, implementing low‐cost screening systems for early detection and effective treatment of PCa at all income levels, and directing targeted subsidies to the health sector, especially to low‐income patients, can help reduce costs for PCa patients.

## 1. Introduction

Globally, prostate cancer (PCa) ranks as the second most frequently diagnosed malignancy and the fifth leading cause of cancer‐related deaths among men [1]. PCa occurs when the prostate gland begins to grow uncontrollably and forms a malignant tumor [2]. The diagnosis of PCa is established through various clinical procedures, including prostate biopsy, measurement of prostate‐specific antigen (PSA) levels, imaging modalities, and transrectal ultrasound (TRUS) [3]. According to the global epidemiological data from the World Health Organization (WHO), 1.4 million men were diagnosed with PCa in 2020, of whom 375,300 died [4, 5].

Additionally, the National Cancer Institute has highlighted that certain risk factors not only promote oncogenesis but also result in elevated treatment expenses and a substantial economic burden [6]. WHO (2020) categorized the factors affecting cancers into behavioral categories (such as tobacco and alcohol consumption and obesity), environmental factors (such as air pollution and exposure to ultraviolet, infrared, and dangerous rays and radiations), infectious factors (such as exposure to some bacteria and viruses, and hepatitis disease), dietary regimes, and other factors such as genetic backgrounds [7]. Regarding PCa, some studies have shown that genetic and environmental factors, including older age, race, diet, obesity, tobacco use, and a positive family history, are influential in its occurrence [4–8].

Cancers impose significant economic and health system costs through reduced productivity, unemployment, and labor losses. Therefore, screening, timely diagnosis, and early treatment of cancers can bring significant health and economic benefits, especially in low‐ and middle‐income countries (LMICs) that have lower cancer survival rates compared to high‐income countries [9]. Studies have ranked PCa as the fourth cancer in terms of imposing health care costs on patients and governments, after lung cancer, breast cancer, and colon cancer [10, 11]. Projections indicate that these costs will continue to rise due to multiple factors, including rising incidence rates, earlier detection practices, management of low‐risk cases, the adoption of costly novel therapies, and overall population growth [12]. Health systems are also seriously concerned about evaluating and controlling the costs associated with diseases and their influencing factors [13]. Examination of underlying factors affecting the high costs of cancer care and their impact on patients is an important research area [14]. Few studies have examined the factors influencing the economic burden of PCa. Research conducted in Canada and Australia (2020), Sweden (2018), and the United States (2004) has identified the following factors as affecting the economic burden of PCa: out‐of‐pocket payments, type of treatment center, commuting for treatment, surgery, length of stay, absence time from work, diagnosis time, and hospitalization [12, 15–17].

Since effective control and reasonable management of escalating healthcare costs play a significant role in reducing disease costs to society, investigating key factors that influence the costs of PCa patients has become an important research topic. The researchers′ search findings indicate that no comprehensive study has examined the influential factors of the direct and indirect costs of PCa, especially in Iran. Therefore, given the importance of cancer‐related costs and their determinants, this study aimed to determine the factors influencing PCa‐related costs among patients referred to diagnostic and therapeutic medical centers in southern Iran.

## 2. Methods

### 2.1. Study Design and Setting

This cost‐of‐illness study employed a bottom‐up, prevalence‐based approach from a societal perspective, focusing on direct and indirect costs. The study was conducted in Fars province, southern Iran, encompassing all healthcare centers providing diagnostic and treatment services for PCa.

### 2.2. Sampling Frame and Procedure

A two‐stage stratified random sampling was employed. Fars province was divided into five strata based on the catchment areas of medical universities providing cancer care: Shiraz, Jahrom, Fasa, Lar, and Gerash. The sample size for each stratum was determined proportionally to the number of registered PCa patients in that stratum. Within each stratum, simple random sampling was used: patient lists were arranged alphabetically, and participants were selected using random numbers generated in *R*.

Patients were included if they [1]: resided in Fars province [2], provided informed consent, and [3] were receiving continuous outpatient or inpatient care for PCa during the study period. Patients were excluded if they withdrew consent or migrated out of the province during data collection.

### 2.3. Sample Size Determination

The required sample size was determined using the findings of Foroughi Moghadam et al. [18], employing the following formula with *S* = 0.48, *d* = 0.083, *α* = 0.05, estimating 127 patients per year for 2021 and 2022, resulting in a total target sample of 254 patients:

(1) n=z1−α/22×s2d2.

### 2.4. Data Collection

Data collection was conducted from March 2020 to March 2022. Direct medical costs (DMCs) were obtained from patients′ medical and financial records, while direct nonmedical costs (DNMCs) and indirect costs (ICs) were gathered through structured interviews with patients or their accompanying companions. The mean values for these cost categories are presented in Table S2. All expenses were initially recorded in Iranian Rials. To enable international comparability, these values were converted to U.S. dollars using the official average exchange rate of 42,000 IRR per USD, as reported by the Central Bank of Iran for the 2021–2022 period.

### 2.5. Identification of Factors Influencing PCa

A scoping review was conducted to identify factors contributing to the economic burden of PCa. This review adhered to the latest Joanna Briggs Institute guidance for scoping reviews, followed a five‐step methodological framework, and reported findings in accordance with the PRISMA‐ScR checklist.

#### 2.5.1. Research Question Formulation

The initial phase of this scoping review aimed to address the following research question: “What are the factors affecting PCa patients’ cost of illness in various countries?”

#### 2.5.2. Study Identification and Selection

The main keywords associated with the research goals were selected by the research team. All relevant research was retrieved using a search strategy. A systematic search for relevant articles was performed across multiple electronic databases, including ISI Web of Science, Scopus, PubMed, ProQuest, Magiran, and SID (Table 1).

**TABLE 1:** Search strategy employed in the study.

Database	Search string	Search results	Limits
Scopus	TITLE‐ABS (neoplasm OR cancer) AND TITLE‐ABS (prostate) AND TITLE‐ABS (cost OR expenditure OR “cost of illness” OR “cost analysis” OR economics OR “burden of illness” OR “economic burden” OR “illness burden” OR “direct cost” OR “indirect cost” OR “financial burden”) AND TITLE‐ABS (factor OR variable OR determinant OR agent OR driver) AND LIMIT‐TO (OA, “all”) AND (LIMIT‐TO (DOCTYPE, “ar”)) AND (LIMIT‐TO (LANGUAGE, “English”))	183	The search was limited to English‐language publications that included at least an abstract in English and included studies available up to December 25, 2021. Keyword searches were conducted within titles and abstracts
PubMed	(((neoplasm [Title/Abstract] OR cancer [Title/Abstract]) AND (prostate [Title/Abstract])) AND (cost [Title/Abstract] OR expenditure [Title/Abstract] OR “cost of illness” [Title/Abstract] OR “cost analysis” [Title/Abstract] OR economics [Title/Abstract] OR “burden of illness” [Title/Abstract] OR “economic burden” [Title/Abstract] OR “illness burden” [Title/Abstract] OR “direct cost” [Title/Abstract] OR “indirect cost” [Title/Abstract] OR “financial burden” [Title/Abstract]) AND (factor [Title/Abstract] OR variable [Title/Abstract] OR determinant [Title/Abstract] OR agent [Title/Abstract] OR driver [Title/Abstract])	291	
WOS	AB = ((neoplasm OR cancer) AND (prostate) AND (cost OR expenditure OR “cost of illness” OR “cost analysis” OR economics OR “burden of illness” OR “economic burden” OR “illness burden” OR “direct cost” OR “indirect cost” OR “financial burden”) AND (factor OR variable OR determinant OR agent OR driver)) AND LANGUAGE: (English) AND DOCUMENT TYPES: (Article) Indexes = SCI‐EXPANDED, SSCI, A&HCI, CPCI‐S, CPCI‐SSH, BKCI‐S, BKCI‐SSH, ESCI Timespan = All years	550	
	TI = ((neoplasm OR cancer) AND (prostate) AND (cost OR expenditure OR “cost of illness” OR “cost analysis” OR economics OR “burden of illness” OR “economic burden” OR “illness burden” OR “direct cost” OR “indirect cost” OR “financial burden”) AND (factor OR variable OR determinant OR agent OR driver)) AND LANGUAGE: (English) AND DOCUMENT TYPES: (Article) Indexes = SCI‐EXPANDED, SSCI, A&HCI, CPCI‐S, CPCI‐SSH, BKCI‐S, BKCI‐SSH, ESCI Timespan = All years	7	
	Combine TI and AB search results with OR	556	
ProQuest	AB (neoplasm OR cancer) AND AB (prostate) AND AB (cost OR expenditure OR “cost of illness” OR “cost analysis” OR economics OR “burden of illness” OR “economic burden” OR “illness burden” OR “direct cost” OR “indirect cost” OR “financial burden”) AND AB (factor OR variable OR determinant OR agent OR driver)	433	
	TI (neoplasm OR cancer) AND TI (prostate) AND TI (cost OR expenditure OR “cost of illness” OR “cost analysis” OR economics OR “burden of illness” OR “economic burden” OR “illness burden” OR “direct cost” OR “indirect cost” OR “financial burden”) AND TI (factor OR variable OR determinant OR agent OR driver)	4	
SID	Cancer N: 2371	After Screen: 14	
	Cancer (in Persian) N: 6929		
Magiran	Prostate cancer N: 718	After Screen: 2	
	Prostate cancer (in Persian) N: 425	After Screen: 15	

#### 2.5.3. Eligibility Criteria

The inclusion and exclusion criteria for this scoping review were established based on the research question and the PCC (population, concept, context) framework to ensure a focused and transparent study selection process.

Population: Studies involving adult men (18 years and older) diagnosed with PCa were included. Studies focusing on other types of cancer or nonhuman subjects were excluded.

Concept: The core concept was the “cost of illness” or “economic burden.” Studies were included if they primarily investigated, identified, or analyzed factors influencing DMCs, DNMCs, or ICs of PCa. Studies that only reported total costs without analyzing influencing factors were excluded.

Context: No geographic restrictions were applied; studies from any country or healthcare setting were considered to capture a broad range of potential cost drivers.

Study design: We included original, peer‐reviewed research articles that used quantitative, qualitative, or mixed‐methods approaches to address the research question. Reviews, letters to the editor, commentaries, conference abstracts, case reports, and unpublished studies (e.g., gray literature, theses) were excluded.

Time frame: The search retrieved all relevant studies published up to the search date (December 25, 2021), with no lower date limit, to provide a comprehensive historical overview of the topic.

Language: To ensure accessibility for the research team, the search was limited to English‐ and Persian‐language (Farsi) articles.

#### 2.5.4. Study Selection

Records retrieved from the aforementioned databases were exported to EndNote software for management and screening. Subsequently, studies meeting the research objectives were selected based on predefined eligibility criteria. Two researchers independently carried out the entire article selection process, and if necessary, a consensus was reached by the third researcher.

#### 2.5.5. Charting the Data

Finally, the information was entered into the data collection form (Table S1 in the Appendix). This table summarizes the selected studies based on factors such as publication year, place (country), participants, and the main results of the studies.

#### 2.5.6. Data Collation, Synthesis, and Reporting of Findings

At this step, information related to factors influencing PCa costs from the selected studies in previous stages was summarized, and the findings were reported.

#### 2.5.7. Integration of Evidence From Scoping Review and Expert Consultation

To develop a comprehensive list of potential cost drivers, a sequential two‐stage approach was employed. Initially, a scoping review was conducted to systematically identify factors reported in the global literature as determinants of PCa costs, providing an evidence‐based foundation for the preliminary list. Subsequently, the preliminary list was presented to a multidisciplinary panel of 10 PCa specialists, including two blood and cancer specialists, two urology oncology fellows, four urology surgeons, and two radiation oncologists. The panel was convened to contextualize the literature findings within the Iranian healthcare setting. Specifically, experts were asked to: (1) identify any additional factors relevant to the local context that were not captured in the scoping review and (2) advise on the availability and feasibility of collecting data on these factors from regional medical records. This process continued until thematic saturation was achieved. The final list of factors (Table 2) represents the union of evidence‐based determinants identified in the international literature and context‐specific factors suggested by local experts. From this final list, variables were selected for data extraction. In the clinical‐pathological domain, histopathological grade (Gleason Score) was selected based on expert recommendations, research team consensus, and consistent documentation in medical records. It was therefore used as the key pathological variable in the analysis. This approach ensured that the selected variables were both well‐informed by existing evidence and pragmatically relevant to the study setting.

**TABLE 2:** Factors influencing costs among PCa patients.

Factors identified through the scoping review	Factors suggested by the specialists
Level of education Radiation therapy Medication regimen Supplementary health insurance coverage Surgical intervention Length of hospitalization Chemotherapy Marital status Comorbidity Out‐of‐pocket payments Healthcare accessibility Commuting for treatment Diagnosis time Inpatient days Physician and specialist visit cost Type of treatment center Patient’s disability due to cancer Surgery Absence time from work Stage Age	Diagnosis imaging services Rehabilitation services Androgen deprivation therapy Medical consultation Chemotherapy side effects Tumor recurrence Laboratory tests Babysitter care costs Work productivity loss Accommodation Patients and their companions’ food Premature mortality due to the disease Monthly income grades

### 2.6. Data Analysis

The list of factors affecting the cost of illness was included in the questionnaire, and the related information was recorded by asking the patient or by reviewing his medical records. Then, considering that our dependent variable (economic burden) is quantitatively continuous and we sought to determine the simultaneous effect of several independent variables on it, the relationship between the factors identified by experts and the economic burden was analyzed using multiple linear regression with a stepwise selection procedure (entry criterion: *p* < 0.05, removal criterion: *p* > 0.10) at a significance level of less than 0.05.

The collected data were analyzed using SPSS version 13.0. Independent *t*‐tests and analysis of variance were employed to identify factors influencing PCa‐related costs. For the variables of premature death, diagnosis time, out‐of‐pocket payments, tumor recurrence, and chemotherapy side effects, which did not have a normal distribution (*p* value < 0.005), the Mann–Whitney test was utilized. Prior to conducting the multiple linear regression analysis, the underlying assumptions were carefully examined. Residual normality was assessed using the Kolmogorov–Smirnov test. Multicollinearity was evaluated using variance inflation factors (VIF), with all values below 5, indicating no significant multicollinearity. Homoscedasticity was examined by plotting residuals against fitted values, which confirmed constant variance. For the sensitivity analysis, a generalized linear model (GLM) with a gamma distribution and a log link was also fitted. Data normality was assessed using the Kolmogorov–Smirnov test. To examine the relationship between continuous variables and healthcare costs, a correlation analysis was performed; the Pearson coefficient was used for normally distributed data, and the Spearman coefficient for non‐normally distributed data. Multicollinearity among independent variables in the final regression model was evaluated using VIF, all of which were below 5, confirming the absence of significant multicollinearity.

## 3. Results

### 3.1. Identified Factors Affecting PCa Costs Through the Scoping Review

**FIGURE 1:**
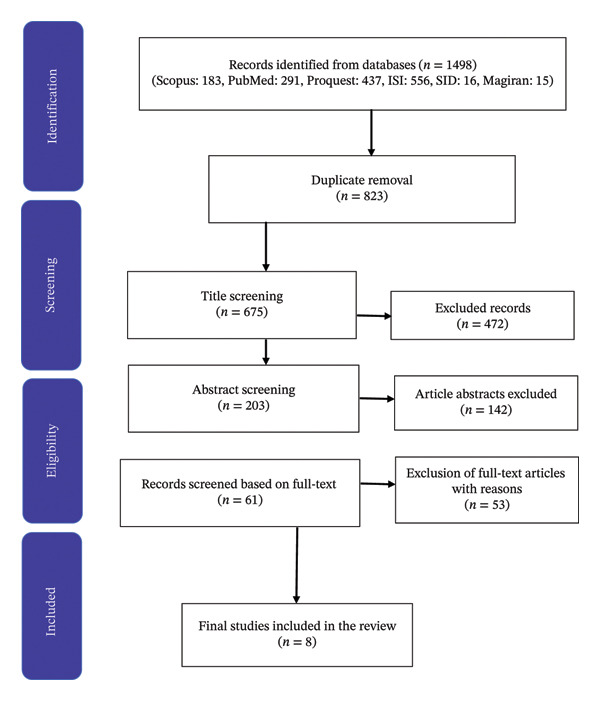
PRISMA flow diagram of the scoping review process.

The results from the relevant databases indicated that 1498 articles matched this study’s field and purpose, of which 675 were duplicates. After screening the titles and abstracts, 472 and 142 articles were removed. Finally, 61 articles were selected for full‐text screening. Ultimately, the researchers selected eight studies that answered this research question (see Figure 1).

The mean age of the participating patients was 69.78 ± 9.40 years. Most of them were married (96.9%), had no academic education (78%), had no premature mortality due to the disease (98.8%), had on‐time diagnosis (88.6%), and did not undergo chemotherapy (81.5%) or radiotherapy (6.77%). Additionally, the majority of patients were in grade 2 of the disease (47.6%), had no comorbidities (52%), had supplementary insurance coverage (58.7%), had no tumor recurrence (81.5%), had a monthly income less than 952 USD (44.9%), and had no chemotherapy side effects (84.6%). Most patients (69.3%) had simultaneously sought both diagnostic and treatment services from private and public centers, and fewer than half (44.1%) had out‐of‐pocket payments for these services (Table 3).

**TABLE 3:** Demographic characteristics of the studied PCa patients.

Factors		Frequency	%	Costs (USD) (mean ± SD)	*p* value
Marital status	Married	246	96.9	14912.631 ± 37417.990	0.770^∗^
	Single (ref)	8	3.1	11038.631 ± 5258.976	

Academic education	Yes	56	22	9909.877 ± 6371.061	0.262^∗^
	No	198	78	16171.026 ± 41506.655	

Premature mortality due to the disease	Yes	3	1.2	248126.174 ± 281712.675	0.007^∗∗∗^
	No	251	98.8	12001.744 ± 8791.613	

Diagnosis time	On‐time (ref)	225	88.6	11855.751 ± 8943.059	0.017^∗∗∗^
	Late	29	11.4	37561.113 ± 104956.848	

Chemotherapy	Yes	47	18.5	28150.672 ± 72680.571	0.001
	No	207	81.5	10568.006 ± 7925.005	

Radiotherapy	Yes	57	22.4	21829.012 ± 11470.398	0.102
	No	197	77.6	12754.125 ± 41177.019	

Grades	I	16	6.3	3030.492 ± 741.303	< 0.001^∗∗^
	II	121	47.6	6662.912 ± 1803.274	
	III	60	23.6	12782.772 ± 1959.809	
	IV	36	14.2	20641.025 ± 2506.435	
	V	21	8.3	66289.18 ± 117285.268	

Comorbidity	Yes	122	48	17340.067 ± 52322.359	0.290^∗^
	No	132	52	12434.304 ± 8977.01	

Out‐of‐pocket payments	Yes	112	44.1	20311.647 ± 54480.401	0.034^∗∗∗^
	No	142	55.9	10435.998 ± 7422.663	

Type of treatment center	Public	78	30.7	21718.478 ± 64806.917	0.179^∗^
	Public–private	176	69.3	11720.312 ± 9114.51	

Covered by supplementary health insurance	Yes	149	58.7	11282.32 ± 8952.869	0.127^∗^
	No	105	41.3	19769.053 ± 56076.592	

Tumor recurrence	Yes	47	18.5	31606.720 ± 82572.890	< 0.001^∗∗∗^
	No	207	81.5	10972.465 ± 8061.188	

Monthly income (USD)	I < 952	114	44.9	14376.042 ± 16533.73	0.268^∗∗^
	II (952–1429)	78	30.7	10194.994 ± 6106.261	
	III (1429–1905)	40	15.7	12827.956 ± 11402.788	
	IV (> 1905)	22	8.7	36800.893 ± 117923.453	

Chemotherapy side effects	Yes	39	15.4	35319.804 ± 90180.535	< 0.001^∗∗∗^
	No	215	84.6	11066.715 ± 8339.133	

**Factors**	**Mean ± SD**	**Spearman correlation coefficient**		**p** **value**	

Distance to the nearest health center	85.40 ± 97.807	0.169		0.007	
Age	69.87 ± 9.404	−0.101		0.110	
Inpatient days	6.72 ± 9.172	0.331		< 0.001	

^∗^ *T*‐test.

^∗∗^ANOVA.

^∗∗∗^Mann–Whitney *U* test.

### 3.2. Factors Associated With PCa

The analysis revealed statistically significant associations between several variables and PCa‐related costs. These included premature mortality due to the disease (*p* = 0.007), time of diagnosis (*p* = 0.017), chemotherapy treatment (*p* = 0.001), tumor grade (*p* < 0.001), disease recurrence (*p* < 0.001), and chemotherapy‐induced adverse effects (*p* < 0.001). Furthermore, Spearman correlation analysis demonstrated that the financial burden on patients and their families was significantly correlated with the mean distance to the nearest healthcare facility (*p* = 0.007) and the duration of hospitalization (*p* < 0.001), as detailed in Table 4.

**TABLE 4:** The association between predictor variables and PCa‐related costs was examined using linear regression analysis.

Factors		Simple regression			Multiple regression		
		Coefficient B	Std. error	*p* value	Coefficient B	Std. error	*p* value
Marital status	Married	3874	13258.367	0.770			
	Single (ref)						

Academic education	Yes	−6261.148	5572.685	0.262			
	No						

Premature mortality due to the disease	Yes	236124.430	15437.844	< 0.001	217659.780	14684.923	< 0.001
	No						

Diagnosis time	On‐time (ref)	25705.362	7100.284	< 0.001			
	Late						

Chemotherapy	Yes	17582.665	5307.320	0.001			
	No						

Radiotherapy	Yes	9074.887	5521.927	0.102			
	No						

Grades	I	13198.774	2031.124	< 0.001	9031.002	1509.110	< 0.001
	II						
	III						
	IV						
	V						

Comorbidity	Yes	4905.763	4625.323	0.290			
	No						

Out‐of‐pocket payments	Yes	9875.649	4623.022	0.034			
	No						

Type of treatment center	Public	−9998.165	4981.135	0.05			
	Public‐Private						

Supplementary health insurance	Yes	−8486.733	4672.661	0.071			
	No						

Tumor recurrence	Yes	20634.254	5820.691	< 0.001			
	No						

Monthly income (USD)	I < 952	3930.303	2377.454	0.100	4472.580	1609.439	< 0.001
	II (952–1429)						
	III (1429–1905)						
	IV (> 1905)						

Chemotherapy side effects	Yes	24253.087	6239.970	< 0.001			
	No						

Factors	Mean ± SD	Simple regression			Multiple regression		
		Coefficient B	Sta. error	*p* value	Coefficient B	Sta. error	*p* value
Distance to the nearest health center	85.40 ± 97.807	0.413	23.726	0.986			
Age	69.87 ± 9.404	−627.079	243.588	0.011			
Inpatient days	6.72 ± 9.172	466.721	251.300	0.064			

### 3.3. Results of Simple Linear Regression Analysis

As shown in Table 4, simple linear regression analysis revealed that the costs of PCa due to premature mortality due to the disease, late diagnosis, undergoing chemotherapy services, increasing cancer grade, tumor recurrence, and the presence of chemotherapy side effects have increased approximately by 236,124 USD, 25,705 USD, 17,582 USD, 13,198 USD, 20,634 USD, and 24,253 USD, respectively (*p* value < 0.001). Seeking treatment at public centers has led to a mean cost reduction of 9998.165 USD (*p* value = 0.05). Additionally, with each year increase in patients’ age, their costs have shown a decreasing trend (*p* value = 0.011) (Table 4).

### 3.4. Multiple Linear Regression Analysis Results

Multiple regression analysis further confirmed a statistically significant association between premature mortality due to PCa and the associated costs. Accordingly, the occurrence of premature mortality due to the disease significantly increased the costs by 217,660 USD (*p* < 0.001). Furthermore, increases in disease grade and income level were associated with higher patient costs of 9031 USD and 4472 USD (*p* < 0.001), respectively (Table 4).

The multiple regression model was statistically significant (*p* < 0.001). Furthermore, the residuals were found to be normally distributed (*p* = 0.93). To assess the robustness of the findings, a sensitivity analysis was performed using a GLM with a gamma distribution and a log link. The results were consistent with the multiple linear regression model, confirming that premature mortality (*p* < 0.001), disease grade (*p* < 0.001), and monthly income (*p* < 0.001) remained significant predictors of PCa costs. In general, the results from conducting independent‐samples *t*‐tests, ANOVA, Mann–Whitney *U* tests, and simple and multiple regression analyses were largely consistent in terms of the significance of the effect sizes, including premature mortality due to the disease, grades, and monthly income. The results also showed that the total mean cost of the disease was 14716.47 USD, with DMCs, DNMCs, and ICs accounting for 46%, 15%, and 39%, respectively. Also, in the DMCs, the largest share of costs was related to hospitalization (33.92%) (Table S2 in the Appendix).

## 4. Discussion

The incidence rate of PCa has been on the rise worldwide in recent years [19]. Despite a decrease in the mortality rate of this disease, costs are expected to increase due to greater diagnosis, earlier detection, and improved patient survival. Until new strategies are developed to enhance the efficiency of healthcare delivery, the costs of PCa will continue to increase [20]. This study aimed to identify the factors affecting the costs of PCa in patients referred to medical centers providing diagnostic and treatment services in southern Iran.

The results of this research, based on simple regression, indicate that premature mortality due to the disease, delayed diagnosis of the disease, undergoing chemotherapy services, increased disease grade, out‐of‐pocket payments by patients, selection of private centers for diagnostic and treatment services, tumor recurrence, chemotherapy side effects, and younger age of patients lead to increased costs for PCa patients.

Multiple regression analysis revealed a statistically significant association between premature mortality due to PCa and disease‐related costs. Patients who experienced premature mortality had much higher costs compared to other patients. This increase can be explained by the fact that premature mortality occurs more frequently in patients at an advanced grade of the disease who receive more complex treatments such as chemotherapy, radiotherapy, and expensive medications, resulting in increased disease costs. Moreover, each premature mortality is considered a cost to society as it leads to the loss of individual productivity during the lost years of life, especially in the working ages, resulting in a substantial increase in indirect costs. Ngcamphalala and colleagues (2022) in South Africa [21], Hao and colleagues (2020) in Sweden [22], and Carter and colleagues (2016) in Australia [23] also identified premature mortality as a factor affecting PCa patient costs, which aligns with the current study’s findings. However, it is worth mentioning that, as stated in some studies, mainly because the disease and death of PCa patients occur at an older age, the premature death rate of these patients is lower than that of other cancer patients [23–25].

According to the findings of this study, cancer grade can also be mentioned as another important factor in increasing disease costs, in such a way that a significant increase in disease grade leads to increased costs, which can be because the overall mean cost in higher disease grades is increased due to the increased use of diagnostic and therapeutic resources, higher hospitalization rates, more visits to cancer clinics, and greater need for home care. In fact, disease grade at diagnosis is an important determinant of treatment costs for patients. Treatment of more advanced, higher‐grade diseases is generally more complex or invasive than treatment for early‐stage disease. Ter Heine and colleagues also showed in their study in the Netherlands (2017) that, as metastasis develops and PCa worsens, disease costs increase [26]. Mojahedian and colleagues, in their study in Iran (2019), show that an increase in disease grade can impose high costs on the healthcare system and patients with PCa. They state that, depending on the stage of treatment and the grade of the disease, in addition to the costs of visits, medicine, tests, imaging, and hospitalization, patients and their families may also have to pay for diapers, specialized diets, toilet seats, electric mattresses, and supplements. Since many PCa patients, especially those with advanced disease, must travel to centers far from their homes for treatment, DNMCs and ICs increase, thereby increasing the mean cost per patient [24]. The results of the review study by Roehrborn and Black on PCa patients across different countries indicated that costs increased from grade I to grade V. The costs for each patient depended on the cancer grade at diagnosis, survival, and the chosen treatment [20]. The study by Torvinen and colleagues in Finland also indicated that treatment for metastatic and higher‐grade disease was significantly more expensive than treatment for early‐stage PCa [27]. The results of these studies are consistent with those of the present study.

Furthermore, the results of the present study showed that household income level is associated with the cost of illness; that is, as household income increased, the cost of illness also increased. This finding may be attributed to the likelihood that patients with higher household income and purchasing power are more inclined to seek private healthcare services and to use more costly transportation and accommodation options, thereby contributing to higher overall illness‐related costs. The study by Kc and colleagues in North Carolina found that the main reason for not receiving healthcare among low‐income men with PCa was their poor social and economic status and lack of access to healthcare [28]. However, contrary to this study’s results, Joyce and colleagues found that higher disease costs were associated with older age and lower family income among PCa patients. A higher number of patients with advanced PCa among low‐income individuals has led to increased costs for this group of men with PCa due to late diagnosis and treatment [29]. Additionally, Ngcamphalala and colleagues in South Africa [21] and Shen and colleagues in a study in the United States during 2011–2013 [30] also showed that PCa patients with low household income were often unemployed and had higher rates of lack of insurance coverage. Low‐income patients generally had more advanced disease and a higher grade at the time of diagnosis, indicating that factors other than treatment, such as income, are the most important factors in deciding to seek treatment in these patients.

Like other studies, the present research had some limitations. One of its limitations stems from patients′ self‐reporting and recall bias regarding DNMCs and ICs. Additionally, incomplete information in some medical records and the non‐cooperation of some patients and their companions with the researchers in providing accurate cost information can be considered as other limitations of this study. Furthermore, the analysis of disease severity relied on histopathological grade, as comprehensive clinical stage (TNM classification) data were not systematically available for the cohort. While grade is a strong and validated predictor, future studies incorporating both grade and stage could provide a more detailed understanding of cost drivers.

## 5. Conclusion

This study identified several factors affecting PCa costs, including premature mortality due to the disease, disease grade, and monthly income level. Based on the results of the present study, patients with advanced or higher‐grade PCa had higher costs. Therefore, the country’s healthcare system should prevent patients from reaching metastatic disease and premature mortality by implementing low‐cost screening systems for early detection and effective treatment at all income levels. Also, directing targeted subsidies toward the health sector, especially to low‐income patients, can help reduce the costs of PCa patients.

**Nomenclature**
CBI: Central Bank of Iran
DMCs: Direct medical cost
DNMCs: Direct nonmedical cost
GBD: Global burden of disease
ICs: Indirect cost
JBI: Joanna Briggs Institute
PCa: Prostate cancer
PSA: Prostate‐specific antigen
TRUS: Trans rectal ultrasound
USD: US dollar
WHO: World Health Organization

## Author Contributions

Ramin Ravangard, Khosro Keshavarz, and Abdosaleh Jafari contributed to the study conception and design. Faride Sadat Jalali and Shahryar Zeighami were responsible for data collection. Ramin Ravangard, Khosro Keshavarz, Abdosaleh Jafari, Mozhgan Seif, and Faride Sadat Jalali performed data analysis. All authors participated in drafting and revising the manuscript.

## Funding

This research received no specific grant from any funding agency in the public, commercial, or not‐for‐profit sectors.

## Disclosure

All authors read and approved the final version of the manuscript.

## Ethics Statement

This study was approved by the Ethics Committee of Shiraz University of Medical Sciences (Ethics Code: IR.SUMS.REC.1400.052). Participation was voluntary, and written informed consent was obtained from all patients. Patients and their companions were assured that their responses would be kept confidential. To ensure ethical compliance and protect patient privacy, all participants were identified by unique codes on data collection forms. The study was conducted in accordance with the principles of the Declaration of Helsinki.

## Consent

The authors have nothing to report.

## Conflicts of Interest

The authors declare no conflicts of interest.

## Supporting Information

Supporting information associated with this article includes the following tables:

Table S1: Detailed information on selected studies.

Table S2: The mean direct medical, direct nonmedical, and indirect costs per studied PCa patient (USD).

## Supporting information


**Supporting Information** Additional supporting information can be found online in the Supporting Information section.

## Data Availability

The datasets generated and analyzed during the current study are available from the corresponding author upon reasonable request.
