# Supplementary material for: Determinants of the Cost of Illness in Iranian Prostate Cancer Patients
Source: Prostate Cancer. 2026 Apr 15;2026:2149782. doi: 10.1155/proc/2149782 (PMC13080342; doi:10.1155/proc/2149782)
Supplement: Supplementary file 1 — Supporting Information Additional supporting information can be found online in the Supporting Information section. [file PROC-2026-2149782-s001.docx]

**Appendix:**

**Table S1. Detailed information on selected studies**

| **Results**  **(The influential factors)** | **Participants** | **Place** | **Year** | **Author** | **Title** | **N** |
| --- | --- | --- | --- | --- | --- | --- |
| Out of pocket payments  Private drug insurance | 171 patients with prostate cancer | Canada | 2020 | Abir El-Haouly | Out-of-pocket costs and perceived financial burden associated with prostate cancer treatment in a Quebec remote area: A cross-sectional study ([1](#_ENREF_1)) | 1 |
| Type of treatment center  Commuting for treatment | 111 patients with prostate cancer | Australia | 2020 | Neli Slavova | Marked variation in out-of-pocket costs for cancer care in Western Australia ([2](#_ENREF_2)) | 2 |
| Chemotherapy  Patient disability due to cancer | 500 patients with prostate cancer | Iran | 2019 | Foroughi Moghadam | Economic Burden of Prostate Cancer in Iran: Measuring Costs and Quality of Life ([3](#_ENREF_3)) | 3 |
| Surgery  Length of stay  Absence time from work | 2638 patients with prostate cancer | Sweden | 2018 | Forsmark | Health economic analysis of open and robot-assisted laparoscopic surgery for prostate cancer within the prospective multicentre LAPPRO trial ([4](#_ENREF_4)) | 4 |
| Primary treatment costs | 2417 patients with prostate cancer | Italy | 2017 | Umberto Restelli | Economic burden of the management of metastatic castrate-resistant prostate cancer in Italy: a cost of illness study ([5](#_ENREF_5)) | 5 |
| Chemotherapy  Marital status | 201 patients with cancer | Iran | 2015 | Bahmei | Examination of medical and non-medical direct costs of outpatients and hospitalized cancer patients in Shiraz ([6](#_ENREF_6)) | 6 |
| Age  Stage  Comorbidity | 42484 patients with prostate cancer | Canada | 2009 | Krahn | Healthcare costs associated with prostate cancer: estimates from a population‐based study ([7](#_ENREF_7)) | 7 |
| Diagnosis time  Hospitalization | 5250 patients with cancer | America | 2004 | Chang | Estimating the cost of cancer: results on the basis of claims data analyses for cancer patients diagnosed with seven types of cancer during 1999 to 2000 ([8](#_ENREF_8)) | 8 |

**Table S2. The mean direct medical, direct non-medical, and indirect costs per studied PCa patient (USD)**

|  | **Type of costs** | **Total**  **Mean** | **% of**  **total costs** |
| --- | --- | --- | --- |
|  |  |  |  |
| **Direct medical costs** | Physicians and oncologist visits | 145.91 | 46 |
|  | Radiation therapy | 1687.43 |  |
|  | Chemotherapy | 288.34 |  |
|  | Diagnosis Imaging Services (DIS) | 943.27 |  |
|  | Cryotherapy | 12.94 |  |
|  | hormone therapy | 653.45 |  |
|  | Laboratory tests | 5.11 |  |
|  | Biopsy | 376.71 |  |
|  | Hospitalization | 2309.99 |  |
|  | Medications and drugs | 386.28 |  |
|  | **Total** | **6809.42** |  |
| **Direct non-medical costs** | Accommodation | 276.42 | 15 |
|  | Transportation of patients and their companions | 594.19 |  |
|  | Patients’ and their companions’ food | 199.15 |  |
|  | Phone and internet calls with family | 4.16 |  |
|  | Purchasing assistive devices | 637.03 |  |
|  | Babysitter and housemaid | 526.81 |  |
|  | **Total** | **2237.75** |  |
| **Indirect costs** | Patient companions’ absenteeism due to patient care | 1837.60 | 39 |
|  | Patients’ absence from work due to the disease | 1318.43 |  |
|  | Premature mortality | 2513.27 |  |
| **Total Cost** | **Total** | **5669.30** | **100** |
|  |  | **14716.47** |  |

1. El-Haouly A, Lacasse A, El-Rami H, Liandier F, Dragomir A. Out-of-pocket costs and perceived financial burden associated with prostate cancer treatment in a Quebec remote area: A cross-sectional study. Curr Oncol. 2020;28(1):26-39.

2. Slavova-Azmanova NS, Newton JC, Saunders CM. Marked variation in out-of-pocket costs for cancer care in Western Australia. Med J Aust. 2020;212(11):525-6.

3. Foroughi Moghadam MJ, Ayati M, Rangchian M, Pourmand G, Haddad P, Nikoofar A, et al. Economic Burden of Prostate Cancer in Iran: Measuring Costs and Quality of Life. Middle East J Cancer. 2019;10(2):139-55.

4. Forsmark A, Gehrman J, Angenete E, Bjartell A, Björholt I, Carlsson S, et al. Health economic analysis of open and robot-assisted laparoscopic surgery for prostate cancer within the prospective multicentre LAPPRO trial. Eur Urol. 2018;74(6):816-24.

5. Restelli U, Ceresoli GL, Croce D, Evangelista L, Maffioli LS, Gianoncelli L, Bombardieri E. Economic burden of the management of metastatic castrate-resistant prostate cancer in Italy: a cost of illness study. Cancer Manag Res. 2017;9(1):789-800.

6. Bahmei J, Rahimi H, saleh Jafari A, Habibyan M. Examination of medical and non-medical direct costs of outpatients and hospitalized cancer patients in Shiraz, Iran. Payesh Health Mon. 2015;14(6):629-37.

7. Krahn MD, Zagorski B, Laporte A, Alibhai SM, Bremner KE, Tomlinson G, et al. Healthcare costs associated with prostate cancer: estimates from a population‐based study. BJU Int. 2010;105(3):338-46.

8. Chang S, Long SR, Kutikova L, Bowman L, Finley D, Crown WH, Bennett CL. Estimating the cost of cancer: results on the basis of claims data analyses for cancer patients diagnosed with seven types of cancer during 1999 to 2000. Clin Oncol. 2004;22(17):3524-30.
